# Melanoma-Derived DNA Polymerase Theta Variants Exhibit Altered DNA Polymerase Activity

**DOI:** 10.1021/acs.biochem.3c00670

**Published:** 2024-04-26

**Authors:** Corey Thomas, Lisbeth Avalos-Irving, Jorge Victorino, Sydney Green, Morgan Andrews, Naisha Rodrigues, Sarah Ebirim, Ayden Mudd, Jamie B. Towle-Weicksel

**Affiliations:** Department of Physical Sciences, Rhode Island College, 600 Mount Pleasant Avenue, Providence, Rhode Island 02908, United States

## Abstract

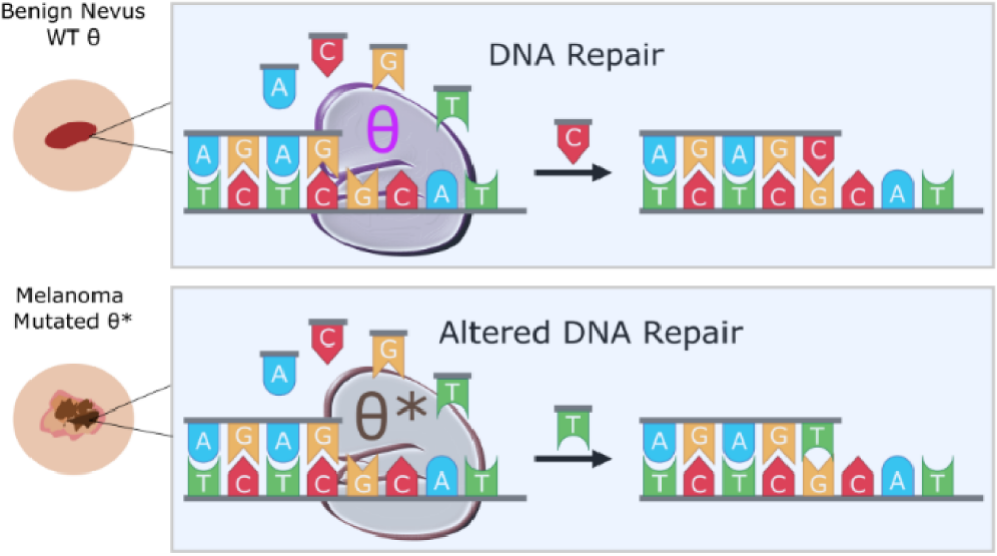

DNA polymerase θ (Pol θ or POLQ) is primarily involved in repairing double-stranded breaks in DNA through an alternative pathway known as microhomology-mediated end joining (MMEJ) or theta-mediated end joining (TMEJ). Unlike other DNA repair polymerases, Pol θ is thought to be highly error-prone yet critical for cell survival. We have identified several POLQ gene variants from human melanoma tumors that experience altered DNA polymerase activity, including a propensity for incorrect nucleotide selection and reduced polymerization rates compared to WT Pol θ. Variants are 30-fold less efficient at incorporating a nucleotide during repair and up to 70-fold less accurate at selecting the correct nucleotide opposite a templating base. This suggests that aberrant Pol θ has reduced DNA repair capabilities and may also contribute to increased mutagenesis. Moreover, the variants were identified in established tumors, suggesting that cancer cells may use mutated polymerases to promote metastasis and drug resistance.

## Introduction

DNA is constantly damaged by endogenous and exogenous factors, including free radicals, chemical agents, and ionizing and UV radiation. Endogenous damage alone is estimated to occur at a rate of at least 20,000 lesions per cell per day.^1^ These lesions result in a variety of issues, including the formation of abasic sites, thymine dimers, single strand breaks, and/or double strand breaks, which, if left unrepaired, can lead to genomic instability, cancer, and/or cell death. Due to this high level of DNA damage, the cell employs DNA repair pathways including homologous recombination and nonhomologous end joining to repair double strand breaks and to maintain the stability of the genome. DNA polymerase theta (Pol θ, protein or POLQ, gene) is the major DNA polymerase in the alternative double-stranded DNA repair pathway known as microhomology-mediated end joining (MMEJ) or theta-mediated end joining (TMEJ).^2−4^ Unlike the more robust and precise repair of double strand breaks in the homologous recombination repair pathway, TMEJ utilizes internal microhomologies of 2–6 base pairs within the DNA it is repairing as a template.^3,5^ Despite Pol θ being a naturally error-prone repair enzyme,^6,7^ it is hypothesized that the TMEJ pathway is important for cell survival as it acts as an auxiliary repair method when other repair pathways are compromised.^8−11^

Overexpression of DNA polymerases has been identified as a negative factor in patient outcomes in a variety of cancers.^12^ Current literature for POLQ confirms similar findings that overexpression is particularly harmful to patients especially those with lung and breast cancers.^13,14^ The mechanism for why overexpression of POLQ is so detrimental to patient outcomes is unclear. In terms of cancer cells, one hypothesis is that the highly mutagenic POLQ allows cancer cells to proliferate and survive any potential chemotherapeutics due to an increased mutagenesis rate.^15^ There is also a link between cells that are deficient in homologous recombination and overexpression of POLQ, which might suggest that the cell is forced into repairing DNA damage through a more error-prone pathway, again to the benefit of a proliferating cancer cell.^8,9^

While overexpression or loss-of-function of POLQ is useful for looking at the importance of the protein at a cellular level, studying the biochemical kinetics of mutant protein will provide insight into the mechanism of POLQ in mutagenesis on a molecular level.^16^ Sporadic and hereditary mutations have been found in all five DNA polymerase families expressed in a variety of tumors.^17−21^ Many of these cancer-associated variants have been characterized *in vivo* and *in vitro* to understand their biochemical and physiological phenotypes. When studied in cell lines, expression of variants leads to cellular transformation, reduced repair, increased amounts of double strand breaks, and chromosomal aberrations, suggesting that variant polymerases have the potential to be cancer drivers.^22−24^ Biochemical studies have suggested that the same DNA polymerase variants often experience slower polymerization rates, increased mutagenesis, and/or poor repair abilities past lesions.^21−23,25−28^ Little is known about the biochemical mechanism of WT Pol θ or the effect a cancer-associated variant has on the overall polymerization rates and mutagenesis. Thus, we wanted to explore the mechanistic function of Pol θ during DNA repair.

**Figure 1:**
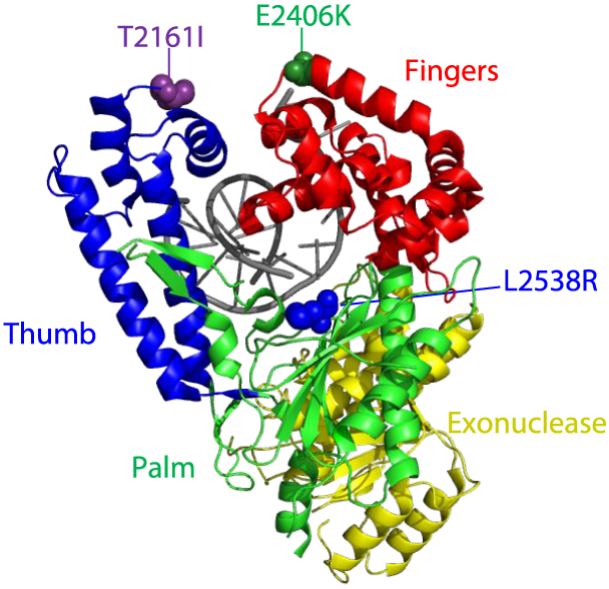
Missense amino acid substitutions found in the polymerase domain of DNA Pol θ. The truncated c-terminal polymerase domain of Pol θ has four subdomains: thumb (blue), fingers (red), palm (green), and exonuclease domain (yellow).^35^ The missense amino acid changes from melanoma patients are represented by spheres with T2161I colored purple, E2406K colored dark green, and L2538R in blue. The DNA substrate is indicated in gray (adapted from protein data bank code 4 × 0Q^35^).

We have identified several POLQ variants from melanoma patients from Tissue Resource Core of the Yale SPORE in Skin Cancer.^29^ Pol θ is a large A-family DNA polymerase (290 kDa) enzyme that contains an N-terminus helicase domain (residues 1–891) and a C-terminal polymerase domain (residues 1819–2590) tethered together by an unstructured central domain (residues 892–1818).^30−36^ The C-terminus can be isolated and characterized as a fully functional DNA polymerase where it contains classic DNA polymerase subdomains (Figure 1) including the DNA binding thumb (residues 2093–2217), nucleotide binding fingers (residues 2333–2474), catalytic active site palm (residues 2218–2590), and an exonuclease-like domain (residues 1819–2090).^36,37^

Focusing specifically on the C-terminal DNA polymerase domain, we selected variants from each of the three subdomains (thumb, fingers, and palm) that were predicted to be detrimental to the enzyme’s function through SIFT and PolyPhen algorithms (Table 1, Figure 1).^38,39^ Mutations were introduced into the isolated C-terminal construct *PolQM1*(37) for an in-depth analysis of deoxynucleotide affinity (*K*_d(dNTP)_) and rates of DNA extension (*k*_pol_). All three variants (T2161I, E2406K, and L2538K) demonstrate decreased fidelity and polymerization kinetics, providing important structural insight into the key residues needed for accurate DNA repair and reduced mutagenesis.

**Table 1:** Missense Amino Acid Substitutions in DNA Polymerase Theta from Melanoma Tumors with Algorithm Predictions^29^ Generated from SIFT and PolyPhen-2

variant	Pol θ subdomain	melanoma type	stage	SIFT	PolyPhen-2
T2161I	thumb	sun-exposed	II	deleterious	probably damaging
E2406K	fingers	ocular	IV	deleterious	probably damaging
L2538R	palm	sun-exposed	IV	deleterious	probably damaging

## Materials and Methods

### Materials

All chemicals and reagents were purchased through Sigma-Aldrich (St. Louis, MO), Bio-Rad Laboratories (Hercules, CA), and AmericanBio (Canton, MA) unless indicated. Oligonucleotides were purchased from Integrated DNA Technologies (Newark, NJ). Oligos for the assays are purified through HPLC with standard desalting. Activities of wild-type (WT) and variants were assayed at a minimum of three replicates, using at least two protein preparations and by at least two individuals.

### Variant Acquisition and Functional Predictions

Melanoma tumor samples were obtained through collaboration with the Specimen Resource Core of the Yale SPORE in Skin Cancer. Sample preparation, nucleic acid extraction, and whole-exome sequencing were collected as previously described.^29,40^ To assess the impact of each amino acid substitution on overall function, we analyzed the full amino acid sequence under default conditions using the algorithms SIFT^38^ and PolyPhen-2,^39^ using default settings.

### Generation of Cancer-Associated Variants

pSUMO3 vector containing truncated C-terminal wild-type human DNA Polymerase θ from amino acid residues 1792–2590 (POLQM1^37^) was generously donated by Dr. Sylvie Doublié from the University of Vermont. Mutations were generated by site-directed mutagenesis on this plasmid using a QuikChange II Site-Directed Mutagenesis Kit (Agilent Technologies). Primers used were as follows:

T2161I:

Forward primer-5′-tgtcaatccctcttctgatagaacccagagttttcttg-3′

Reverse primer-5′-caagaaaactctgggttctatcagaagagggattgaca-3′

E2406K:

Forward primer-5′-gagagcagatgggcattaaaaaaaatgatgctgcatgcta

Reverse primer-5′-tagcatgcagcatcattttttttaatgcccatctgctctc

L2538R:

Forward primer-5′-atataggagttcatcatggcgttgaaggatgaagaagcc

Reverse primer-5′-ggcttcttcatccttcaacgccatgatgaactcctatat

Mutated plasmids were verified through sequencing through the Molecular Informatics Core at the University of Rhode Island.

### Molecular Modeling

PyMol 1.3^41^ was used to generate a representation of the C-terminal POLQM1 and its cancer-associated variants from the crystal structure as previously described.^35^

### Expression and Purification of WT Pol θ and Its Cancer-Associated Variants

Expression and purification of plasmids containing human wild-type (WT) and/or its variants as previously described according to Hogg et al.,^37^ with the following modifications to optimize for maximum DNA polymerase activity. Plasmid was transformed into Rosetta2(DE3) pLysS competent cells (EMD Millipore) and plated on LB agar plates containing 100 μg/mL of ampicillin and 34 μg/mL of chloramphenicol. Due to an observed toxicity to the *E. coli* cells during overexpression of human Pol θ, we followed the expression protocol developed by Hogg et al. that yielded the greatest amount of soluble, well-folded protein for kinetic studies.^37,42^ Colonies were directly inoculated into 1 L of autoinduction Terrific Broth (0.5% w/v glycerol, 0.05% w/v dextrose, 0.2% alpha-lactose, 1 M potassium phosphate buffer at pH 7.0, 100 μg/mL ampicillin, and 34 μg/mL chloramphenicol) and incubated for 60 h at 20 °C. Cells were harvested by centrifugation at 5K RPM for 10 min, and pellets were stored at −80 °C.

Pellets were thawed on ice and resuspended in Lysis buffer (20 mM Tris, pH 7; 300 mM NaCl; 0.01% NP-40; 10% glycerol; 20 mM Imidazole; 5 mM β-mercaptoethanol (βME); 0.120 mM PMSF; and EDTA-free Protease Inhibitor Mixture (Roche Applied Science)) and sonicated for 6–8 rounds for 30 s. Soluble cell fractions were separated via centrifugation at 15K RPM for 30 min. Soluble protein fractions were separated by fast protein liquid chromatography (FPLC) with an imidazole gradient by mixing binding buffer A (20 mM Tris, pH 7; 300 mM NaCl; 0.01% NP-40; 10% glycerol; 20 mM Imidazole; and 5 mM βME) with elution buffer B (buffer A with 500 mM Imidazole) on a 5 mL His-Trap FF Crude Nickel Column (GE Healthcare). Fractions containing Pol θ were pooled and were separated further on a 5 mL HiTrap Heparin HP (GE Healthcare) with a NaCl gradient by mixing binding buffer C (20 mM Tris, pH 7; 300 mM NaCl; 0.01% NP-40; 10% glycerol; and 5 mM βME) and elution buffer D (buffer C with 2 M NaCl). Pooled fractions containing Pol θ were cleaved overnight at 4 °C with SUMO2 protease (Fisher Scientific). Cleaved protein was separated by a 5 mL HiTrap Chelating HP (GE Healthcare) column using an imidazole gradient by mixing binding buffer E (20 mM Tris, pH 7; 300 mM NaCl; 0.01% NP-40; 10% glycerol; 10 mM imidazole; and 5 mM βME) with elution buffer B. Cleaved Pol θ was collected in the flow-through fraction with the 6xHIS-sumo remaining on the chelating column. The imidazole was removed from the protein preparation by a final HiTrap Heparin column by mixing buffer C and buffer D and omitting NP-40 detergent. Cleaved, purified protein (yield approximately 10 μM) was rapidly frozen in liquid nitrogen and stored at −80 °C for approximately 3 months.

### Generation of DNA Substrate

The duplex DNA substrate was generated using two oligodeoxynucleotides (IDT). The primer (5′6-FAM label) was annealed to the complementary 40-mer template as described below:^43^

5′-/FAM/TTTGCCT TGA CCA TGT AAC AGA GAG

CGGA ACT GGT ACA TTG TCT CTC GCA CTC ACT CTC TTC TCT

Annealing was verified by a 12% native PAGE with annealed and primer only samples and scanned on an RB Typhoon scanner (Cytiva) with an FAM fluorescence filter.

### Circular Dichroism

To compare secondary structure of WT and Pol θ variants, the ellipticity of 3 μM of protein in 10 mM K_2_HPO_4_ buffer were measured from 190 to 280 nm at room temperature (20 °C). Samples were measured in a 0.2 cm quartz cuvette in a J-815 CD Spectropolarimeter (Jasco, Brown University).

### Primer Extension Assay

Pol θ (750 nM) was preincubated with 50 nM duplex DNA for 5 min at 37 °C. Nucleotide (correct, incorrect, or all) were added at a final concentration of 50 μM and incubated together with complex Pol θ and duplex DNA for an additional 5 min at 37 °C. Reactions were stopped with an 80% formamide-EDTA solution, and products separated on a 20% denaturing polyacrylamide gel. The gel was scanned on an RB Typhoon scanner with a FAM fluorescence filter.

### Rapid Chemical Quench Assays

Pol θ (100 nM) was preincubated on ice with 300 nM 5′FAM-labeled DNA substrate and rapidly mixed with 100 μM dCTP (correct nucleotide) and 10 mM MgCl_2_ in a reaction buffer (20 mM Tris HCl, pH 8.0, 25 mM KCl, 4% glycerol, 1 mM βME, and 80 μg/mL BSA) using an RQF-3 Chemical Quench Flow apparatus (KinTek) at 37 °C from 0 to 0.6 s. Reactions were stopped by the addition of 0.5 M EDTA and collected into microcentrifuge tubes containing 90% formamide sequencing dye. Products were separated on a 15% denaturing polyacrylamide gel and scanned on a RB Typhoon scanner as described above. The extended products (*n* + 1) were quantified using ImageQuant software and fit to a nonlinear regression full biphasic burst equation^44^ (eq 1) ± standard deviation using Prism 9 GraphPad Software:
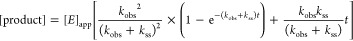
1

For burst kinetics, multiple replicates were as follows: WT *n* = 19 replicates, 10 different protein preparation; L2538R *n* = 16 replicates, 9 different protein preparations; E2406K *n* = 17 replicates, 5 different protein preparations; T2161I *n* = 13 replicates, 7 different preparations. Each burst assay was completed by three individuals.

### Single-Turnover Kinetics

Pol θ and 5′FAM-labeled DNA substrate were assayed at a 4:1 ratio, as determined by the active site and empirical enzyme titrations (Supporting Information Methods). Correct nucleotide, dCTP, was titrated from 0 to 1000 μM with 50 nM DNA substrate and 200 nM Pol θ from 0 to 0.6 s on the RQF at 37 °C. Incorrect nucleotides were titrated from 0 to 1000 μM for 0 to 300 s with the same Pol θ and DNA concentrations by hand at 37 °C. Products were separated on a 15% denaturing polyacrylamide gel, scanned, and quantified as described above. Data were fit to a single exponential equation (eq 2) to determine *k*_obs_, which is the observed rate at each dNTP concentration (±standard deviation).
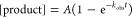
2

The *k*_obs_ was plotted against nucleotide concentration for each of the 4 deoxyribonucleotides and fit to the hyperbolic equation ± standard error (eq 3):
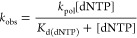
3

For single-turnover kinetics, multiple replicates were as follows: WT *n* = 20 replicates, 5 different protein preparation; L2538R *n* = 10 replicates, 3 different protein preparations; E2406K *n* = 4 replicates, 3 different protein preparations; T2161I *n* = 5 replicates, 2 different preparations. Each single-turnover assay was completed by two individuals.

## Results

### Twelve Percent of Melanoma Tumors Contained Mutations in POLQ

Of the 250 melanoma samples obtained from the Yale SPORE in Skin Cancer, 29 patients had at least one mutation in the POLQ (11.6% occurrence), with 9 having missense mutations in the C-terminal polymerase domain, defined between amino acids (AA) 1792–2590.^37^ To identify key residues that are involved in nucleotide incorporation and fidelity, we choose a cancer-associated variant from each of the three major DNA polymerase subdomains (thumb, fingers, and palm) and where the nonsynonymous substitution was predicted to be detrimental to the function of the enzyme by the SIFT and PolyPhen-2 algorithms (Figure 1, Table 1).

**Figure 2:**
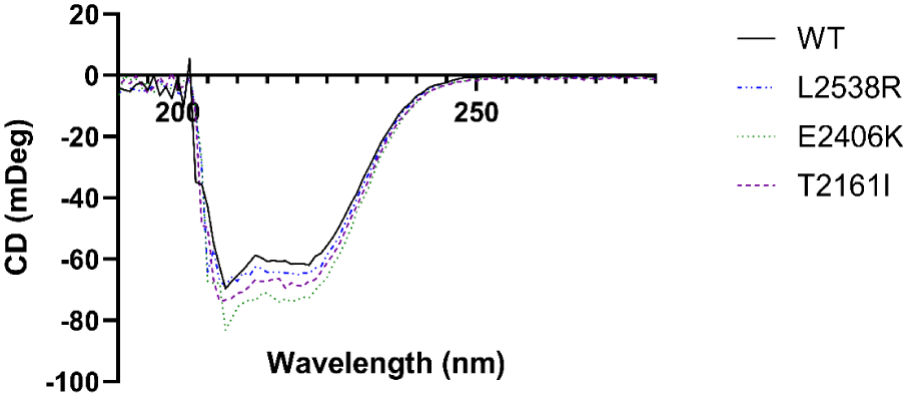
Secondary structure of cancer-associated variants similar to that of WT Pol θ. Circular dichroism spectra of 3 μM WT (black, solid line) and variants L2538R (blue, dot and dashed line), E2406K (green, small dashed line), and T2161I (purple, large dashed line) in 10 mM K_2_HPO_4_ scanned from 190 to 280 nm at 20 °C.

The T2161I variant was originally discovered in a tumor from patient with stage 2 melanoma (Figure 1, Table 1) Interestingly, this mutation is located in insert 1 of the thumb domain, an area that is highly flexible and not resolved in the crystal structure (Figure 1).^35^ E2406K was a stage IV ocular melanoma mutation located in the fingers subdomain, and the palm domain variant L2538R came from a melanoma patient with stage IV sun-exposed melanoma (Figure 1, Table 1). All three variants were predicted to be potentially deleterious in function via prediction algorithms. In addition, we performed a Clustal Omega sequence alignment of the C-terminal end of human Pol θ with Pol θ from *Mus musculusandDanio Rerio* (Figure S1).^45^ Amino acids T2161, E2406, and L2538 were identical across species, suggesting that these may be important functional residues for Pol θ. We also compared the polymerase domain of human Pol θ to two additional A-family DNA polymerases, human Pol ν and Klenow fragment. L2538 was conserved, but Pol ν and the Klenow fragment lacked the T2161 as they do not contain the Insert 1 motif unique to Pol θ. In addition, there was no similarity for the Glu at position 2406 across Pol ν and the Klenow fragment. The mutations were introduced individually via site-directed mutagenesis into the human pSUMO3 vector containing the C-terminal POLQM1.^37^ Constructs were expressed and purified from *E. coli* with an average final concentration of 10 μM. To ensure that individual point mutations did not affect the overall structure, circular dichroism spectroscopy was performed on WT and variants at 20 °C. The spectra of each variant were similar to that of WT Pol θ, suggesting that all variants had similar secondary structure characteristics (Figure 2).

### Cancer-Associated Variants Bind to Duplex DNA Substrate Similar to WT

To evaluate the affinity of WT and cancer-associated variants for the duplex DNA substrate, we performed an electrophoretic mobility shift assay (EMSA). Pol θ was titrated against the 5′-FAM-labeled duplex DNA substrate, and complexes were observed on a native PAGE (Figures S2, S3). The apparent equilibrium dissociation constant *K*_D(DNA)_ was determined to be similar among WT and all of the cancer-associated variants with WT value of 18 ± 3 nM, T2161I 13 ± 3 nM, E2406K 14 ± 3 nM, and L2538R 18 ± 4 nM.

### Pol θ Variants Are Able to Extend Duplex DNA

**Figure 3:**
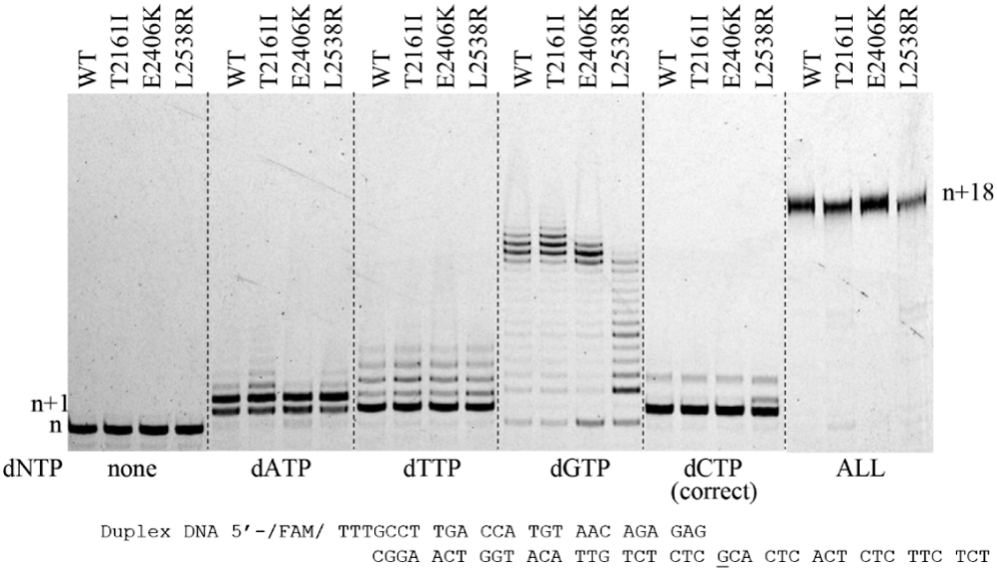
DNA Pl θ and its cancer-associated variants can fully extend duplex DNA. Pol θ and variants (750 nM) were preincubated with duplex DNA (50 nM) at 37 °C for 5 min. Samples were mixed with 50 μM dNTP (as indicated above) and incubated for 5 min at 37 °C. Samples were separated on a 15% denaturing polyacrylamide gel and visualized on an RB Typhoon scanner. The gel was scanned as described in the Materials and Methods section using an RB Typhoon scanner.

To assess the overall DNA polymerase activity, all variants and WT Pol θ were assayed for initial DNA polymerase activity with 750 nM Pol θ (variant or WT) preincubated with 50 nM duplex DNA along with 50 μM dNTP for 5 min at 37 °C (Figure 3).

Duplex DNA 5′-/FAM/TTTGCCT TGA CCA TGT AAC AGA GAG

CGGA ACT GGT ACA TTG TCT CTC GCA CTC ACT CTC TTC TCT

We looked for the incorporation of the nucleotide opposite template G (underlined in Figure 3) and potential further extension after the initial nucleotide insertion event. Incorrect nucleotide insertion and extension for WT and the cancer variants were similar for G:dATP and G:dTTP repeatedly adding dATP to position *n* + 4 and a similar *n* + 5 was seen with dTTP. Adding dGTP was the most prolific with WT, T2161I, and E2406K, creating a repeating sequence of 16 to 17 dGs. L2538R appeared to be able to extend the primer terminus to 14–15 dGTPs, but there was more of a stalled effect with product buildup at corresponding template C, especially at positions *n* + 2 and *n* + 6. Incorporating the correct dCTP nucleotide appeared to occur at the *n* + 1 position, with a darker band appearing at this position as well as a band *n* + 4 for all enzymes. Interestingly, L2538R had a predominate band at *n* + 2, which would be incorporation opposite the adjacent template C. When all dNTPs were provided, we observed full extension to a 43-mer product with a 5 min incubation.

The promiscuous extension results observed in this assay highlighted a distinctive ability to incorporate multiple dGTP to almost a full-length product under these assay conditions. We hypothesized that Pol θ had a sequence context specificity that afforded the DNA polymerase the ability to skip bases and insert and extend dGTP when presented with alternating dCTP in the template. To test this, we altered the DNA template such that all of the dCs were replaced with dGs (Figure S4). Within that sequence context, we observed reduced extension for both the WT and variants with dGTP, creating only the *n* + 2 product. L2538R was further hindered by this sequence, extending only to *n* + 1, highlighting the importance of the templating DNA sequence. Incorporation of dTTP with WT and variants on the altered template behaved similarly to the original DNA template but did extend an additional two nucleotides. We expected to see a similar extension pattern with dATP, but surprisingly, it too only extended to *n* + 1 product despite having multiple dTs in the template. Correct nucleotide (dCTP) extension was observed as expected with WT and variants, and we observed increased extension, presumably due to greater dG content in the template.

### Pol θ Variants Experience Biphasic Burst Kinetics

**Figure 4:**
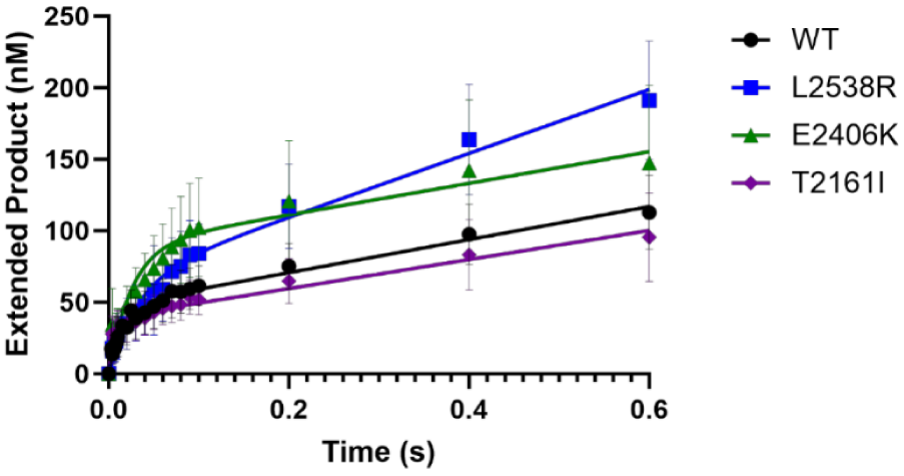
Wild-type and cancer-associated variants experience fast biphasic burst kinetics. Presteady state burst kinetics were performed by preincubating 300 nM duplex DNA with 100 nM WT (circles), T2161I (diamond), E2406K (triangles), or L2538R (squares) and reacting with 100 μM dCTP (correct) from 0.0037 to 0.6 s. Data were graphed as extended product versus time and fit to eq 1 (±standard deviation). Results are from multiple replicates (error bars; *n* = 13–19) and at least 5 different protein preparations assayed at 37 °C.

To further explore the rate nucleotide incorporation as well as the misincorporation and exaggerated extension of Pol θ and the variants we observed in the previous qualitative experiment, we assayed the enzyme under a time-based, presteady state burst kinetics (Figures 4, S5 and Table 2) using the aforementioned duplex DNA substrate (CG template) in excess (see Materials and Methods). Under this specific condition, the DNA substrate is in excess (300 nM) over the enzyme (100 nM). DNA extension products were plotted over time and fit to a full burst equation (eq 1), allowing us to define two distinct first-order rate constants that are typical of most DNA polymerases: (1) a rapid observed burst rate (*k*_obs_) that is initial product formation; and (2) a slower linear second-rate indicative of product release (*k*_ss_).^46^ WT experienced a *k*_obs_ of 60 s^–1^ and a rate limiting product release rate (*k*_ss_) of 2.3 s^–1^ consistent with previous presteady state studies.^35,37^ Variant T2161I experienced a similar *k*_ss_ rate of 2.5 s^–1^ compared to that of WT but had a polymerization rate that was twice as fast (*k*_obs_) at 142 s^–1^ on the same DNA substrate. Variants L2538R and E2406K experienced a 2-fold slower *k*_obs_ compared to the WT at 28 and 38 s^–1^, respectively. The *k*_ss_ for E2406K was calculated at 1.2 s^–1^ and for L2538R at 3.1 s^–1^. Despite the different *k*_obs_ rates for all variants, it was clear that the rate limiting step of product release was consistent with WT and other DNA polymerases, but DNA polymerase activity prior to product release is altered.

**Table 2:** Observed Correct dCTP Incorporation Rates of Pol θ and Cancer-Associated Variants (±Standard Deviation)

Pol θ	*k*_obs_ (s^–1^)	*k*_ss_ (s^–1^)	*E*_app_ (nM)	fold change^a^
WT	60.0 ± 6.1	2.3 ± 0.2	51.0 ± 1.8	1.0
T2161I	142.0 ± 22.5	2.5 ± 0.3	41.0 ± 1.8	0.4
E2406K	38.1 ± 5.2	1.2 ± 0.2	95.0 ± 4.8	1.6
L2538R	28.0 ± 5.3	3.1 ± 0.2	80.0 ± 7.0	2.1

a *k*_obs_ WT/ *k*_obs_ variant.

### Cancer-Associated Variants Experience a Different Kinetic Pathway Compared to WT

In order to investigate the difference between the observed DNA polymerization rates between WT and variants, we explored single-turnover kinetics to directly define polymerization rate (*k*_pol_) and apparent equilibrium dissociation constant (*K*_d(dNTP)_) for each nucleotide opposite a DNA template G. Unlike presteady state burst assays, these experiments utilized enzyme concentrations in excess over DNA with varying nucleotide concentrations. For each concentration, an observed rate was defined corresponding to the direct turnover of the substrate to product (*k*_pol_) without the steady-state products obscuring the rates.^46^ Similar to the presteady state burst assay, the enzyme and duplex DNA were preincubated prior to the introduction of nucleotide to ensure the binary complex was formed, and we were monitoring the chemistry of nucleotide incorporation. For single-turnover experiments, the actual Pol θ/duplex DNA concentration needs to be determined through an active site titration. Duplex DNA was titrated from 0 to 300 nM against 100 nM Pol θ and 100 μM dCTP. The amount of product formed over time was graphed using the full burst equation (eq 1) to determine amplitude (*E*_app_) value for each DNA concentration. These data were fit to a quadratic equation (Supplemental eq 2) to determine the percentage of active sites for the DNA/enzyme complex.^47^ This was repeated for each individual protein preparation, and experimental protein concentrations were adjusted to equal active protein/DNA complex levels (Figures S6 and S7). Rates and affinity were verified empirically through enzyme titration to ensure maximum product formation (Supporting Information; data not shown). We determined the ratio of 4:1, protein to DNA, provided the maximum product under excess Pol θ conditions.

**Figure 5:**
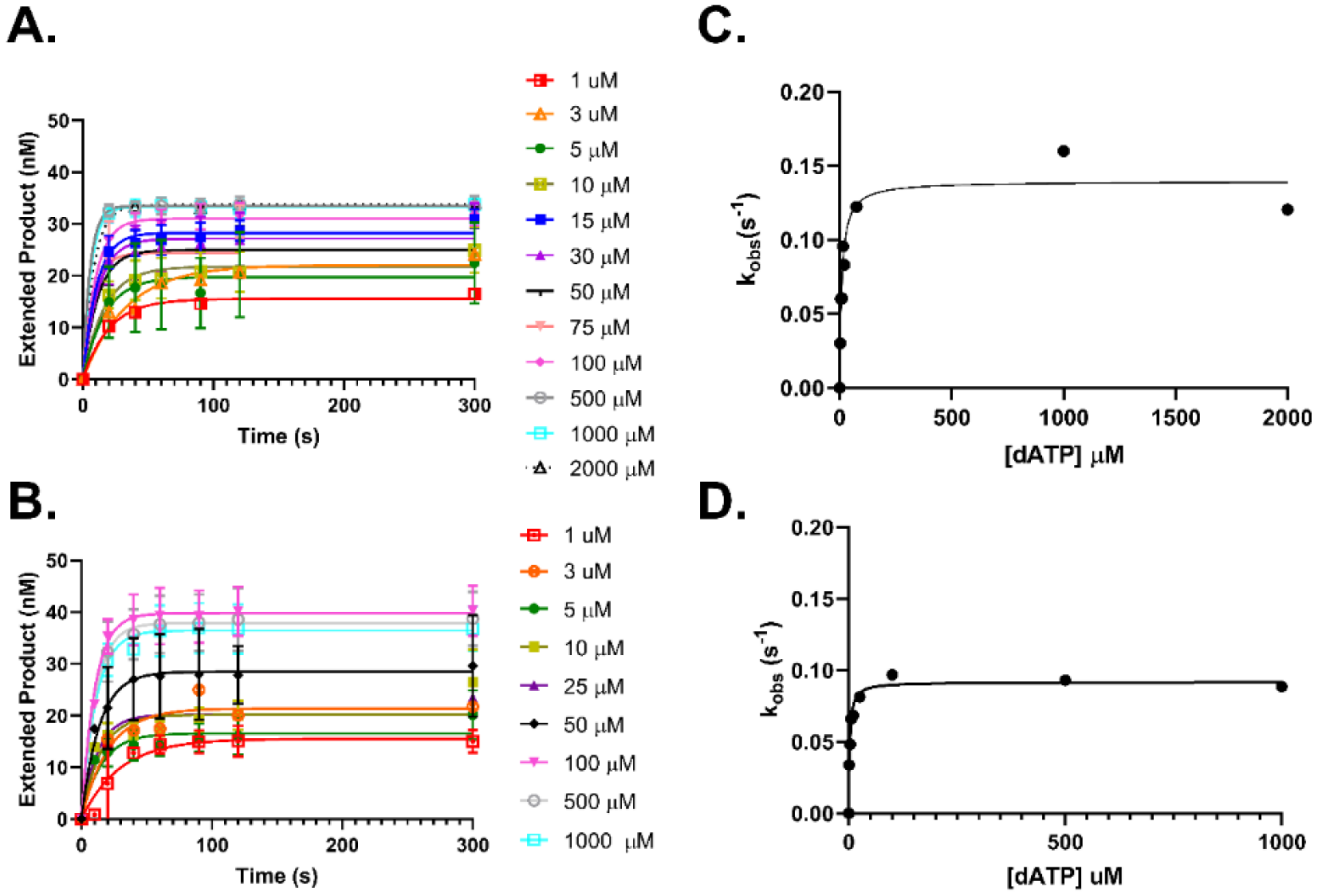
Cancer-associated variants bind to incorrect nucleotides with greater affinity than WT. A representative plot of single-turnover experiments with incorrect dATP opposite template G. Increasing concentrations of dATP were titrated against 50 nM DNA substrate and 200 nM Pol θ (A) or L2538R (B). Extended product was graphed versus time to determine *k*_obs_ (eq 2; ±standard deviation). Results are from multiple replicates (error bars; *n* = 4–20) and at least 2 different protein preparations assayed at 37 °C.The observed rate was plotted against concentration of dATP (C and D) and data fit to a hyperbolic eq 3 to determine *k*_pol_ and *K*_d(dNTP)_ (±standard error of fit).

Through our single-turnover experiments, we observed that all variants had a decrease in fidelity, especially for incorrect incorporation of dATP and dTTP opposite template G (Figures 5 and S8 and Table 3). WT and T2161I demonstrated a rapid polymerization rate for correct (dCTP) nucleotide incorporation of around 167 s^–1^. Both variants E2406K and L2538R experienced reduced correct incorporation *k*_pol_ rates compared to WT with 89 and 41 s^–1^, respectively. All of the *k*_pol_ rates for WT and the variants during incorrect incorporation were considerably reduced to around 0.1 to 0.2 s^–1^ compared to the rapid correct *k*_pol_ rate, although E2406K and L2538R experienced less of a reduction in incorrect *k*_pol_ rates compared to WT and T2161I. Most striking, the *K*_d(dNTP)_ was drastically different for the variants compared to WT. WT experienced a tighter binding affinity for the correct nucleotide, whereas the variants experienced a reduction in affinity to the correct nucleotide by up to 40-fold. The *K*_d(dNTP)_ for incorrect nucleotides, especially for T2161I and E2406K, are much lower compared to correct signifying a higher affinity. These kinetic values revealed a similar reduction in efficiency for the variants with T2161I being 40-fold, E2406K being 30-fold, and L2538R being 10-fold less efficient at incorporating the correct nucleotide (Table 3). Moreover, we observed a reduction in fidelity of the variants compared to WT especially for G:dATP and G:dTTP pairing. T2161I and E2406K were the least faithful compared to WT when incorporating dTTP with 80-fold and 40-fold reduction, respectively. L2538R was the most promiscuous with dATP incorporation opposite template G with a 34-fold reduction in fidelity compared to WT. WT and variants all experience a decrease in *K*_d(dNTP)_ for dGTP binding, especially WT where we observed a *K*_d(dNTP)_ value of 2 μM compared to 5 μM correct. We attribute this to potential DNA template slippage due to the sequence of the DNA template being flanked by dCTP.

**Table 3:** Single-Turnover Kinetics for WT Pol θ and Cancer-Associated Variants on Duplex DNA with Template G^a^

Pol θ	sequence	*k*_pol_ (s^–1^)	*K*_d(dNTP)_ (μM)	Δ*k*_pol_^b^	Δ*K*_d(dNTP)_^c^	efficiency^d^ μM^–1^ s^–1^	Δefficiency^e^	*F*^f^ (×10^2^)	Δfidelity^g^
WT	G:dC	167 ± 5	5.20 ± 0.7			32			
WT	G:dG	0.114 ± 0.004	1.60 ± 0.4			7.1 × 10^–2^		4.5	
WT	G:dA	0.139 ± 0.009	9.71 ± 2			1.4 × 10^–2^		22	
WT	G:dT	0.150 ± 0.01	15.3 ± 4			9.8 × 10^–3^		33	
T2161I	G:dC	167 ± 10	199 ± 40	1	40	0.84	40		
T2161I	G:dG	0.223 ± 0.01	44.3 ± 10	1	30	5.0 × 10^–3^	10	1.7	3.0
T2161I	G:dA	0.133 ± 0.008	15.2 ± 4	1	2	8.7 × 10^–3^	2	1.0	23
T2161I	G:dT	0.111 ± 0.005	5.50 ± 1	1	0.4	2.0 × 10^–2^	0.5	0.4	77
E2406K	G:dC	89.0 ± 5	93.1 ± 20	2	20	0.96	30		
E2406K	G:dG	0.132 ± 0.006	17.4 ± 4	1	10	7.6 × 10^–3^	10	1.3	4.0
E2406K	G:dA	0.160 ± 0.009	33.8 ± 6	1	3	4.7 × 10^–3^	3	2.0	11
E2406K	G:dT	0.120 ± 0.007	10.2 ± 3	1	1	1.2 × 10^–2^	1	0.8	40
L2538R	G:dC	41.2 ± 1	15.70 ± 1.4	4	3	2.6	10		
L2538R	G:dG	0.117 ± 0.004	3.50 ± 0.71	1	2	3.3 × 10^–2^	2	0.8	6.0
L2538R	G:dA	0.0920 ± 0.003	2.29 ± 0.36	2	0.2	4.0 × 10^–2^	0.4	0.7	34
L2538R	G:dT	0.125 ± 0.008	12.08 ± 3.0	1	1	1.0 × 10^–2^	1	2.5	13

a Kinetic rates and constants derived from single-turnover experiments for WT and cancer-associated variants (±standard error).

b WT/variant.

c Variant/WT.

d *k*_pol_/*K*_d_ (μM–^1^s^–1^).

e Efficiency_WT_/efficiency_variant_.

f *F* = (efficiency_correct_ + efficiency_incorrect_)/efficiency_incorrect_.

g Fidelity_WT_/fidelity_variant_.

To test this hypothesis of slippage, a phenomenon that has been seen with other DNA polymerases,^35,48^ we replaced the 5′ template C with a subsequent A (Figure S9). This resulted in a defined *K*_d(dNTP)_ for WT of 21.4 μM, which is a 13-fold decrease in affinity for dGTP with an AG versus a CG template (Figure S9 and Table S2). The variant E2406K also experienced a 2.3-fold loss in affinity for dGTP with this template, but T2161I increased its affinity for dGTP with the AG template by half, with a new *K*_d(dNTP)_ value of 22.5 μM compared to 44.3 μM with the CG template. The *K*_d(dNTP)_ for dGTP with the L2538R variant only increased slightly to 5.1 μM with the AG template and experienced an overall greater preference with the lowest *K*_d(dNTP)_ value of all WT and variants.

Discrimination between correct and incorrect nucleotide selection for both WT and variants can be observed at the level of *k*_pol_ as shown by the slower rate of incorrect incorporation (Table S1). However, at the level of *K*_d(dNTP)_ binding, there is a notable loss in discrimination for the variants compared to WT. For example, T2161I has a 100-fold difference in discriminating against incorrect dTTP incorporation compared to WT. E2406K has a 30-fold loss in discrimination for dTTP as well with L2538R experiencing a greater than 10-fold loss in discrimination of dATP misincorporation opposite template G. Taken together, these results suggest that each of these residues T2161, E2406, and L2538 are important for nucleotide selection and overall fidelity, and the nucleotide selection process for these variants are altered.

## Discussion

**Scheme 1:**
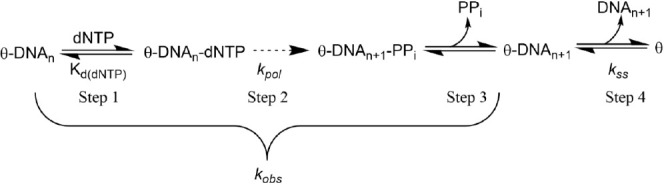
Adapted Pol θ Biochemical Mechanism^60,69^ DNA polymerase θ adopts a similar biochemical mechanism of most DNA polymerases. After DNA binding, DNA polymerases select a nucleotide to match the templating base (step 1), where a conformational change aligns the nucleotide within the catalytic active site for step 2. Once phosphodiester bond is formed, the pyrophosphate from the incoming nucleotide is released (step 3), the product is released (the rate limiting step, step 4), and the next round of nucleotide incorporation can begin.

A DNA polymerase’s biochemical kinetics can provide key insight into DNA damage repair capability. The fidelity, or ability, of the DNA polymerase to select the correct nucleotide during nucleotide incorporation is an important step in DNA repair and preventing genomic instability and cancer. By determining the *k*_pol_ and *K*_d(dNTP)_, a more cohesive mechanism of polymerase activity can be observed, providing insight into how aberrant enzymes may alter this activity.^46^ DNA polymerase θ has been shown to be a low fidelity enzyme that is critical for alternative double strand break repair especially in BRCA-depleted cells.^35,49,50^ What is unclear about the role of Pol θ in DNA repair is whether the polymerase stabilizes the genome through repair or is detrimental to the cell by increasing mutagenesis. Perhaps reinforcing this apparent contradiction, prior studies have explored the role of POLQ in genomic stability in either loss of function or overexpression analyses, which have notable detrimental secondary effects.^8,11^ Additionally, prior to this study, catalytic mutations, without direct connection to disease, were examined in both the N-terminal Helicase and C-terminal polymerase domains for functional studies.^4,35,43,51−53^ Here we report for the first time three Pol θ missense mutations found in melanoma patients that biochemically display altered protein function, suggesting a potential role in genomic instability. These three mutations were strategically selected to explore the subdomains of the C-terminal polymerase domain of Pol θ in order to explore the functional role of each subdomain in a disease context. We generated these mutations *in vitro* and utilized classical primer extension assays to analyze nucleotide selection and incorporation compared to WT to gain insight into the variants overall DNA repair capabilities. A common nucleotide incorporation mechanism for DNA polymerases can also be applied to Pol θ based on our biochemical data (Scheme 1). Pol θ experiences a rapid *k*_obs_ around 60 ± 6 s^–1^ (steps 1–3 including nucleotide binding, polymerization, and pyrophosphate release) that proceeds the slower rate limiting *k*_ss_ rate, step 4 (Figure 4, Table 2). Most kinetic studies of Pol θ have been performed under steady-state conditions,^49^ which can underestimate the more transient nucleotide selection step that occurs just prior to polymerization.^46^ This is the first study to our knowledge that explores the DNA polymerase activity of Pol θ using single-turnover conditions, which allows for direct determination of *k*_pol_ and *K*_d(dNTP)_. Through this approach, we observed typical DNA polymerase activity for WT with it efficiently incorporating the correct dCTP nucleotide opposite template G (Figure 5, Table 3). There was not a considerable decrease in incorrect nucleotide affinity, but the data are consistent with the steady-state assumption that Pol θ is a low fidelity enzyme.^35,48^

In the same way, we observed this low fidelity activity with our initial primer extension assay, which qualitatively explored whether WT and its variants were able to extend duplex DNA with either each nucleotide individually or all together (Figure 3). Interestingly, WT and its variants were able to extend at least 3 nucleotides past the initial template DNA with both correct and incorrect nucleotide. Incorporation and subsequent extension from the template with dGTP were especially apparent generating near full product extension. Moreover, this overextension with dGTP has not been observed in previous studies with Pol θ under steady-state conditions with this DNA template^43^ or with Pol α and Pol β with similar CXC repeating elements in the DNA template.^54^ Why Pol θ under single-turnover conditions could readily incorporate dGTP and extend with the same nucleotide was unclear; however, through changing the provided sequence to remove dCTP from the template strand revealed that preference for dGTP extension within that specific sequence context (Figure S4), a phenomenon that has been previously reported in other DNA polymerases,^55−57^ and potentially could be linked to the enzyme’s ability to bypass certain DNA damage.^7,32,37,58^ Further study of the efficiency and fidelity of Pol θ within specific sequence contexts is needed. Taken together with our single-turnover kinetics, Pol θ experiences a faster polymerization rate than some higher fidelity DNA polymerases, Pol β, γ and ε, but is much less efficient at correct nucleotide insertion.^59−62^

### T2161I Thumb Domain Mutation

**Figure 6:**
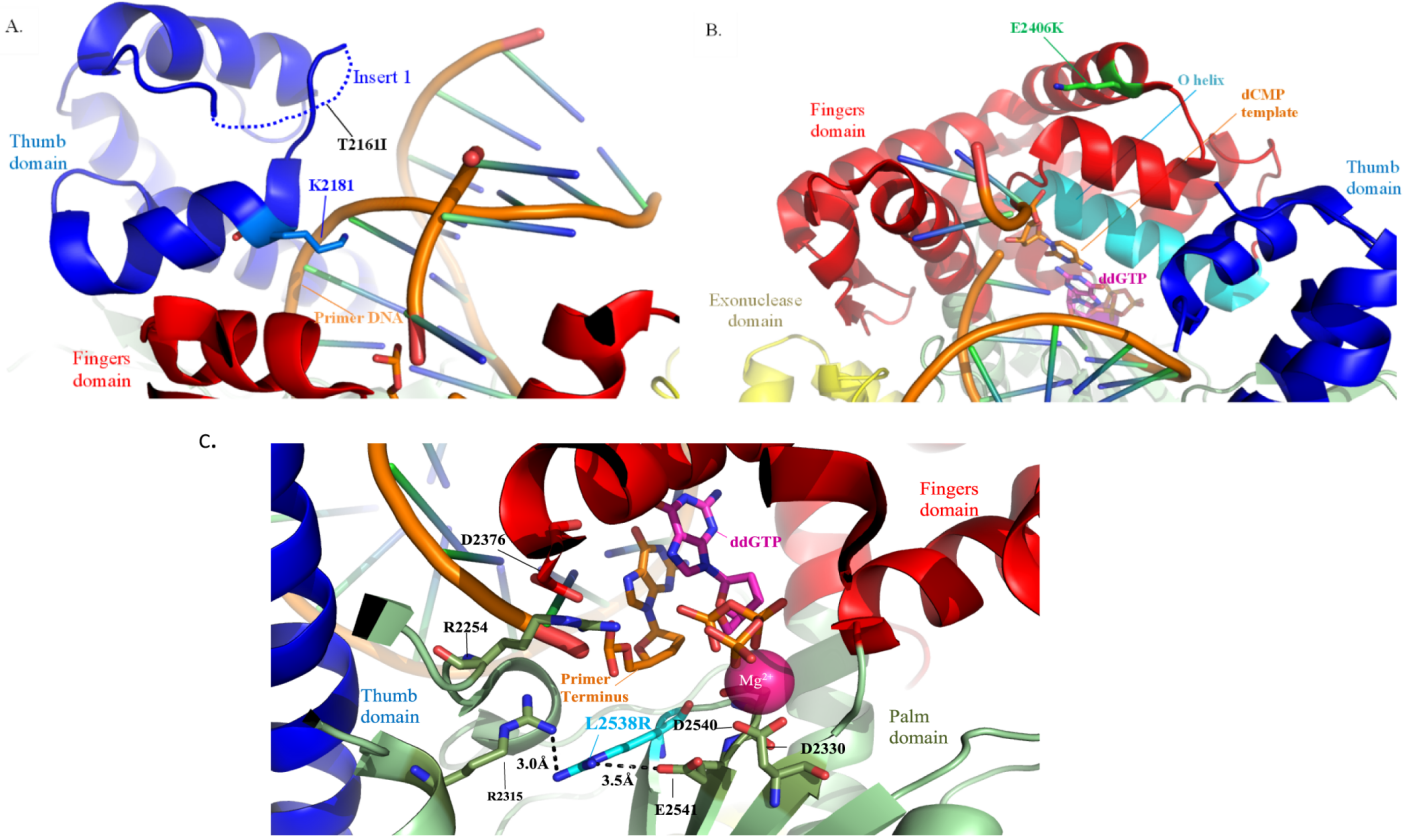
Model of amino acid changes in the cancer-associated variant Pol θ. (A) The thumb domain variant, T2161I, is located in the undefined dashed Insert I (blue dashes). Potential perturbations in hydrogen bonding could take place between Primer DNA (orange with green/blue bars) and/or residue K2181, which interacts with the phosphate backbone of the Primer DNA. (B) Zoomed in structural analysis of the area surrounding the fingers domain variant, E2406K, located in the helix just above the O helix (cyan), which contacts the incoming ddGTP nucleotide aligning it within the dCMP template, suggesting potential structural perturbations. (C) A model zoomed into the active site highlighting the change from a leucine to an arginine at position 2538 (cyan), which makes potential contacts with critical residues including R2315, E2541, and surrounding residue network, which supports the primer terminus (orange), Mg^2+^ (magenta), and incoming ddGTP (magenta). Variant residues were made using the mutagenesis function in PyMOL to replace original amino acids found in Pol θ structure (protein data bank code 4 × 0Q^35^)

The T2161I variant experienced a polymerization rate equally as fast as WT.^35^ Although the Insert 1 region (P2144–F2177) is thought to be involved in processivity,^35,37^ we did not observe any effects on DNA binding (Figure S2), the rate of polymerization (Table 3), or ability to continue to synthesize for the T2161I variant (Figure 3). Instead, there was a large reduction in nucleotide affinity for correct nucleotide selection with a *K*_d(dNTP-correct)_ value of 200 μM compared to 5 μM for WT. Moreover, it appears that T2161I variant prefers and most efficiently incorporates a dTTP opposite G. This could be best explained by the hypothesis that certain mismatched pairs of bases, especially T and G, form alternative hydrogen bonding in a way to promote mutagenesis.^63,64^ We observed that T2161I is less affected by slippage compared to WT as the *K*_d(dNTP)_ value with an AG template is half the value with a CG template, suggesting a greater affinity for dGTP without the C template (Figure S9, Table S2). Why we observe this effect with this thumb variant is unclear. The mutation of an isoleucine from a threonine did not affect the global structure; there was no increase in the insoluble fraction during protein purification, indicating misfolding and protein yields were similar to WT. The location of this variant within the Insert 1 domain (Figure 6a) allows for speculation that subtle changes are made in the microenvironment of Insert 1, most logically important to the primer DNA phosphate backbone interaction. The loss of the hydrogen bonding from the threonine could impact residue K2181, which does coordinate the phosphate backbone of the primer DNA. Although mutational studies looking at the bypass ability of K2181A on damaged DNA suggest reduced activity compared to WT,^35^ our observations with T2161I could suggest that we have uncovered a unique role for Insert 1 in coordinating correct nucleotide selection and incorporation. A cancer cell could likely take advantage of a mutated DNA repair polymerase that retains fast polymerization and introduces more mutations, likely aiding in cancer cell survival and metastasis. Further studies involving different DNA substrates and DNA damage could shed light on the unusual increased mutagenesis rate in this thumb domain variant.

### E2406K Fingers Domain Mutation

The E2406K variant presented similar kinetic behaviors to the other cancer-associated variants (Figure 5, Table 3). The polymerization rate was observed to be different than that of WT with about a 2-fold reduction. Although it was not as striking as the T2161I variant, E2406K did have an 18-fold reduction in affinity for correct, dCTP, selection compared to WT. Like the other variants, its preference was to mismatch dTTP or dATP with template G. The *K*_D(DNTP)_ value associated with dGTP mimics the behavior of WT slippage, but to a lesser extent (Figure S9, Table S2). Located in the finger domain, it seems intuitive that there would be a reduction in nucleotide selection. However, the specific location is far away from any of the critical residues including the highly conserved O-helix residues that interact with the incoming nucleotide including the phosphate interacting R2379 and K2383 and base interacting residues Q2384 and Y2387. In fact, the mutation appears to be at the apex of the fingers domain helical bundle running parallel to the O-helix (Figure 6B). Given its position and the change in charge from a negative to positive, this could cause a change in the intermolecular forces in the O-helix, disrupting the alignment of the incoming nucleotide with the templating base.^65^ The altered DNA polymerase activity and lack of nucleotide selectivity of E2406K are unexpected based on its location but highlight a novel important residue outside of the O-helix that we show to influence fidelity and mutagenesis. Interestingly, the E2406K variant came from a tumor that also had BRCA1 and BRCA2 mutations, suggesting that the cell’s DNA repair capabilities could be further challenged due to a mutagenic Pol θ.^29,66^

### L2538R Palm Domain Mutation

The palm variant L2538R had a reduced observed polymerization rate of 27 s^–1^, which is half of what was observed with WT. Under single-turnover conditions, it remained the slowest of all the variants. Despite this, the affinity for nucleotides was similar to that of WT, with only a slight reduction in overall *K*_d(dNTP)_ for correct dCTP and was the most efficient of the variants with only a 12-fold reduction in efficiency compared to that of WT. This variant had a higher affinity for dGTP compared to correct dCTP, and unlike WT, it was unaffected by slippage. Qualitatively, we observed that the L2538R variant had more difficulty extending past *n* + 1 when the DNA template lost the repeating CXCXCXC pattern compared to WT and the other variants, suggesting that it is more sensitive to the specific DNA sequence (Figures 3 and S4). The *K*_D(DNTP)_ value for dGTP with the AG template was similar to the CG:dGTP value, suggesting that regardless of the sequence context of the template, L2538R still had a higher affinity for dGTP compared to correct dCTP (Figure S9, Table S2). In addition, L2538R did not follow the normal mutagenic G:dTTP mismatch but instead preferred G:dATP pairing, which has been shown to be energetically unfavorable.^63,67^ Unlike the other two cancer-associated variants, the L2538R variant is located in close proximity to the conserved catalytic residue E2541 and subsequent D2330 and D2540 (Figure 6C). This suggests that the new positive charge from the mutated arginine could greatly affect Mg^2+^ metal binding and the incoming nucleotide (ddGTP in the figure) within the active site, potentially perturbing the charge network set in place by the catalytic triad.^35^ In addition, L2358 is located below the sugar ring of the primer terminus, but a residue change to another arginine could perturb the network of arginines at positions 2254 and 2315 that interact with the phosphate backbone of the primer DNA as well as D2376 in the O-helix of the fingers domain, thus slowing down the catalytic process and/or promoting the mispairing of dATP and dGTP through misalignment of the 3′OH for phosphodiester bond formation within the active site.^68^

In summary, we have biochemically characterized three melanoma-derived mutations in human DNA Polymerase theta. We have demonstrated that these variants have reduced affinities for correct nucleotide incorporation and a preference for incorrect nucleotide selection compared to the WT. These data potentially indicate how mutated Pol θ may be more beneficial to tumors and carcinogenesis than WT Pol θ. These data also provide evidence for critical residues that are important for Pol θ activity that impact overall DNA repair and genomic stability. Taken together, it could be hypothesized that the tumor utilizes mutagenesis through aberrant Pol θ to promote genomic instability and further evade cancer therapeutics. Through our biochemical analysis of the activity of mutant Pol θ, we gained a better understanding of how these variants play a role in cancer.

## Supporting Information Available

Additional experiments and methods available in Supporting Information including Supporting methods EMSA; active site titration; empirical enzyme titration; Figure S1: sequence alignment; Figure S2: EMSA; Figure S3: representative polyacrylamide gel for EMSA; Figure S4: primer extension on alternative DNA substrate; Figure S5: representative polyacrylamide gels for presteady state burst kinetics; Figure S6: representative active site titration; Figure S7: representative polyacrylamide gel for active site titration; Figure S8: representative single-turnover polyacrylamide gel; Figure S9: single-turnover with AG DNA template; Table S1: Discrimination of nucleotides; Table S2: AG DNA template summary (PDF)
