# Supplementary material for: Melanoma-Derived DNA Polymerase Theta Variants Exhibit Altered DNA Polymerase Activity
Source: Biochemistry. 2024 Apr 26;63(9):1107–17. doi: 10.1021/acs.biochem.3c00670 (PMC11080051; doi:10.1021/acs.biochem.3c00670)
Supplement: Supplementary file 1 — bi3c00670_si_001.pdf [file bi3c00670_si_001.pdf]

SUPPORTING INFORMATION

Melanoma-derived DNA polymerase theta variants exhibit altered DNA polymerase activity

Corey Thomas, Lisbeth Avalos-Irving, Jorge Victorino, Sydney Green, Morgan Andrews, Naisha Rodrigues, Sarah Ebirim, Ayden Mudd, and Jamie B. Towle-Weicksel*

Department of Physical Sciences, Rhode Island College, 600 Mount Pleasant Avenue, Providence, RI 02908

*Corresponding author's email address: jtowleweicksel@ric.edu

List of materials included: Supporting Methods, Figures S1-9, Tables S1-2, references

SUPPORTING METHODS

Electrophoretic Mobility Shift Assay (EMSA)

Pol θ was titrated from 0-1000nM against 10nM 5-FAM labeled DNA substrate in binding buffer (10 mM Tris HCl, pH 7.5, 6 mM $MgCl_2$, 100 mM NaCl, 10% glycerol, and 0.1% NP-40) and incubated for 1 hour at 25°C. After incubation, samples were mixed with loading dye (25 mM Tris HCl, pH 7.8, 10% glycerol) and loaded on a 6% Native PAGE running at 300V at 4°C[1]. The gel was scanned on an RB Typhoon scanner (Cytiva) with the FAM fluorescence filter. Separated bound and unbound products were quantified using ImageQuant and percent protein bound to DNA was plotted against the log concentration of protein. The $K_{D(DNA)}$ was estimated from the equation:

$$Y = \left[\frac{(mx)}{(x + K_D)}\right] + b$$

Supplemental Equation 1

Active Site Titration

The DNA substrate was titrated from 0 to 300nM against 100nM Pol θ, 100μM dCTP, 10mM $MgCl_2$ in reaction buffer at 37˚C from 0-0.6s. Reactions were stopped by the addition of EDTA and scanned on the Typhoon as described above. Products were quantified and data fit to Equation 2. The E_{app} was plotted against concentration of DNA. The concentration of Active Sites and $K_{D(DNA)}$ were determined by fitting data to a quadratic equation[2].

$$Y = 0.5 \times (K_{D(DNA)} + ActiveSites + X) - (0.25 \times (K_{DNA} + ActiveSites + X)^2 - (AcitveSite \times X))^{0.5}$$

Supplemental Equation 2

An active site titration was performed on each individual protein preparation to determine the active concentration for subsequent single turnover experiments.

Empirical Enzyme Titration

To ensure single turnover conditions, a primer extension assay was performed, DNA substrate (50nM) was reacted with varying concentrations of Pol θ (50nM to 500nM) with 100μM dCTP, 10mM $MgCl_2$, in reaction buffer. Products were separated on a 15% denaturing polyacrylamide gel and scanned on an RB Typhoon as described above. Data was fit to a single exponential equation (Equation 2).

Thumb Domain

```
                                                            Insert 1
H.sapiens Polθ          AKLDAIETQAYQLAGHSFSFTSSDDIAEVLFLELKLPPNREMKNQGSKKTLGSTRRGIDN     2167
M.musculus Polθ         AKLDAIETQAYQLAGHSFSFTSADDIAQVLFLELKLPPNGEMKTQGSKKTLGSTRRGNES     302
D.rerio Polθ            AKLSALESQAYQLAGHSFSLTSPEDVAEVLFLELKLPPNGDLNGLKNKKTLGYTRR---A     369
H.sapiens Polν          ARLKELEQEAHFVAGERFLITSNNQLREILFGKLKLHLLSQRN----------------S     502
Klenow fragment         LRLAELEKKAHEIAGEEFNLSSTKQLQTILFEKQGIKPLKKT------------------     279
                         :*   :* :*: :**. * ::* .::  :** :  :    .
```

Fingers Domain

```
H.sapiens Polθ          YGMGAKSLGEQMGIKENDAACYIDSFKSRYTGINQFMTETVKNCKRDGFVQTILGRRRYLP    2451
M.musculus Polθ         YGMGAKSLGEQMGIKENDAASYIDSFKSRYKGINHFMRDTVKNCRKNGFVETILGRRRYLP    585
D.rerio Polθ            YGMGAKSLGEQMGIEENDAACYIETFKSRYNGIQNFLRETVQKCGKNGYVKTLLGRKRFLP    648
H.sapiens Polν          YGAGKERLAACLGVPIQEAAQFLESFLQKYKKIKDFARAAIAQCHQTGCVVSIMGRRRPLP    746
Klenow fragment         YGMSAFGLARQLNIPRKEAQKYMDLYFERYPGVLEYMERTRAQAKEQGYVETLDGRRLYLP    503
                        ** .    *.  :.:  ::*  ::: : .:*  : .:    :  :. . * * :: **:  **
```

Palm Domain

```
                             Insert 3            Motif 5
H.sapiens Polθ          LSRKRKLQGMFCPIRGGFFILQLHDELLYEVAEEDVVQVAQIVKNEMESA-------VKL     2568
M.musculus Polθ         LLPKRKLKGMFCPMRGGFFILQLHDELLYEVAEEDVVQVAQIVKNEMECA-------IKL     776
D.rerio Polθ            IRLGGRHRNQFRPLRGGYFILQLHDELIYEVAEEDVIQVAQIVKREMESV-------VKL     757
H.sapiens Polν          ----------------ARLVAQIHDELLFEVEDPQIPECAALVRRTMESLEQVQALELQL     838
Klenow fragment         ----------------VRMIMQVHDELVFEVHKDDVDAVAKQIHQLMENC-------TRL     587
                                            :: *:****::** . ::   *  ::. **         :*
```

Figure S1. Cancer associated variants of DNA Polymerase θ are conserved residues in other species. Clustal Omega sequence alignment of the C-terminus of human Pol θ compared to Pol θ from *M. musculus* and *D. rerio* as well as from other A-family DNA polymerases including human Pol ν and Klenow fragment. Each subdomain (thumb, fingers, or palm) of human Pol θ was aligned with the full-length amino acid sequence and the region surrounding the location of the cancer-associated variants is shown here. The red letters (denoted by *) are identical residues; blue (denoted by :) are conserved; purple (denoted by*) are semi-conserved; the boxed letters represented identical amino acids in all 5 sequences. The position of the cancer associated variants are highlighted in green. Structural themes are lined and described above.

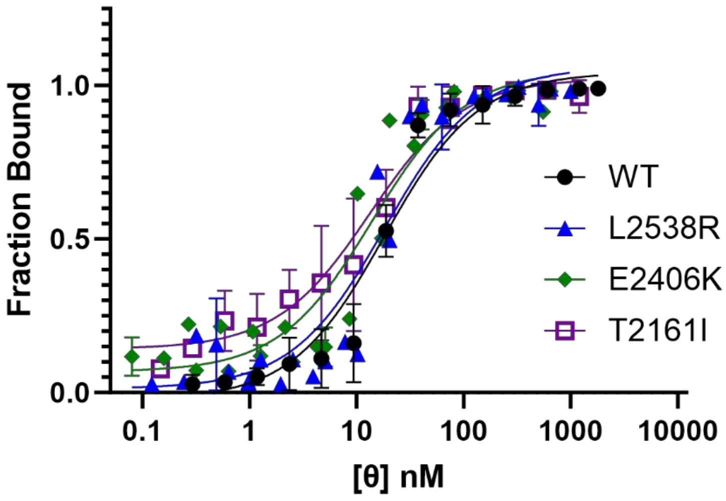

Figure S2. Pol θ cancer variants have a similar dissociation constant for duplex DNA as WT. Pol θ was titrated against 10 nM duplex DNA and incubated for 1 hour at room temperature. Products were separated on a non-denaturing polyacrylamide gel and analyzed using ImageQuant. Fraction bound was calculated and plotted against varying concentrations of Pol θ to determine the $K_{D(DNA)}$ (WT 18 ± 3 nM; T2161I 13 ± 3 nM; E2406K 14 ± 3 nM; L2538R 18 ± 4 nM)

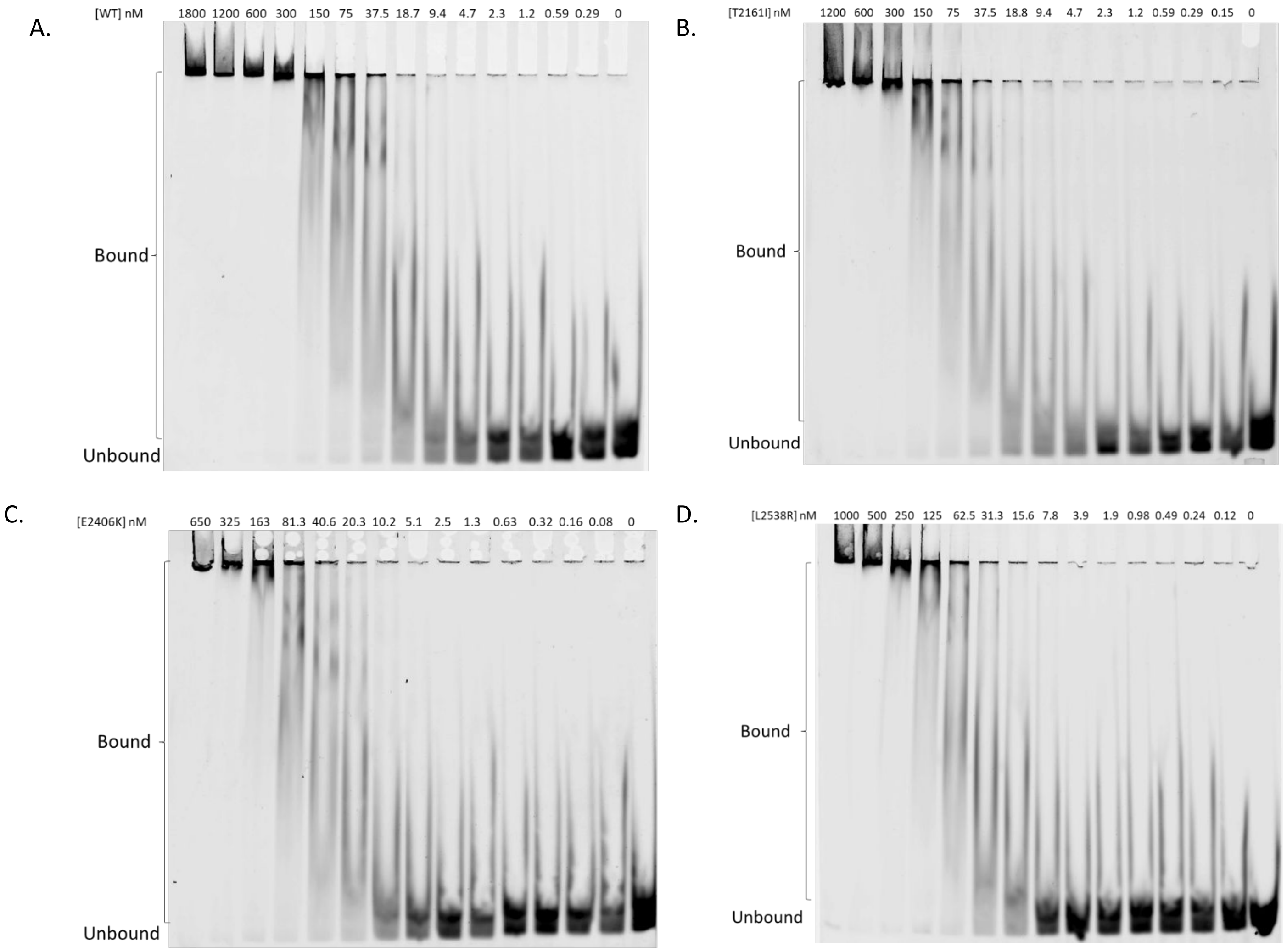

Figure S3. Representative 6 % native polyacrylamide gels for WT and cancer variants. Varying concentrations of WT (A), T2161I (B), E2406K (C), or L2538R (D) were titrated against 10 nM duplex DNA and incubated for 1 hour at room temperature. Products were separated on a 6% non-denaturing polyacrylamide gel visualized on an RB Typhoon scanner with a FAM filter.

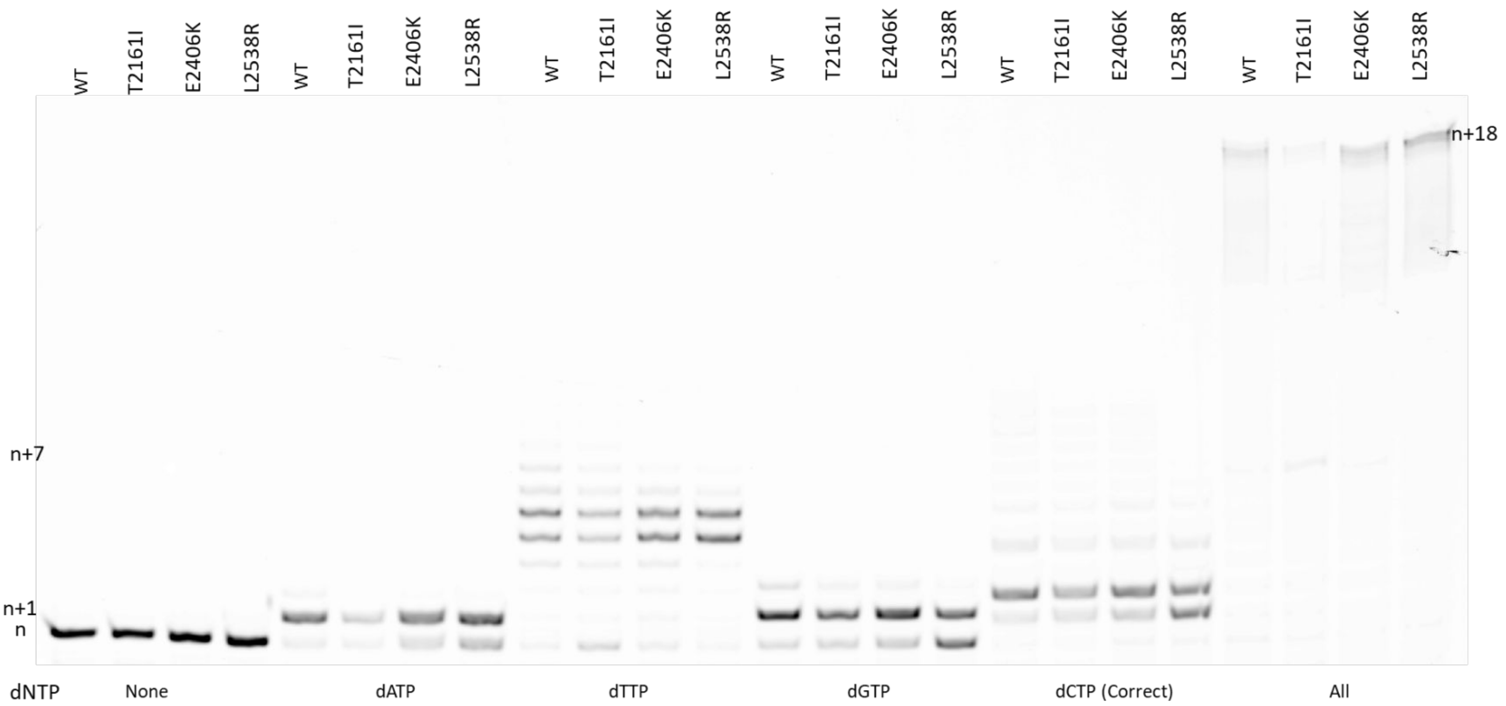

5’FAM/ TTTGCCT TGA CCA TGT AAC AGA GAG
CGGA ACT GGT ACA TTG TCT CTC GAA GTG AGT GTG TTG TGT

Figure S4. DNA Pol θ and its cancer-associated variants demonstrate sequence context-specificity. Pol θ and variants (750 nM) were preincubated with 50 nM duplex DNA that were dG rich with repeating elements of GXG (highlighted in red). Samples were combined with 50 µM dNTP as indicated above. Products were separated on a 15% denaturing polyacrylamide gel and visualized on an RB Typhoon scanner.

```
Duplex DNA 5'-/FAM/ TTTGCCT TGA CCA TGT AAC AGA GAG
                        CGGA ACT GGT ACA TTG TCT CTC GCA CTC ACT CTC TTC TCT
```

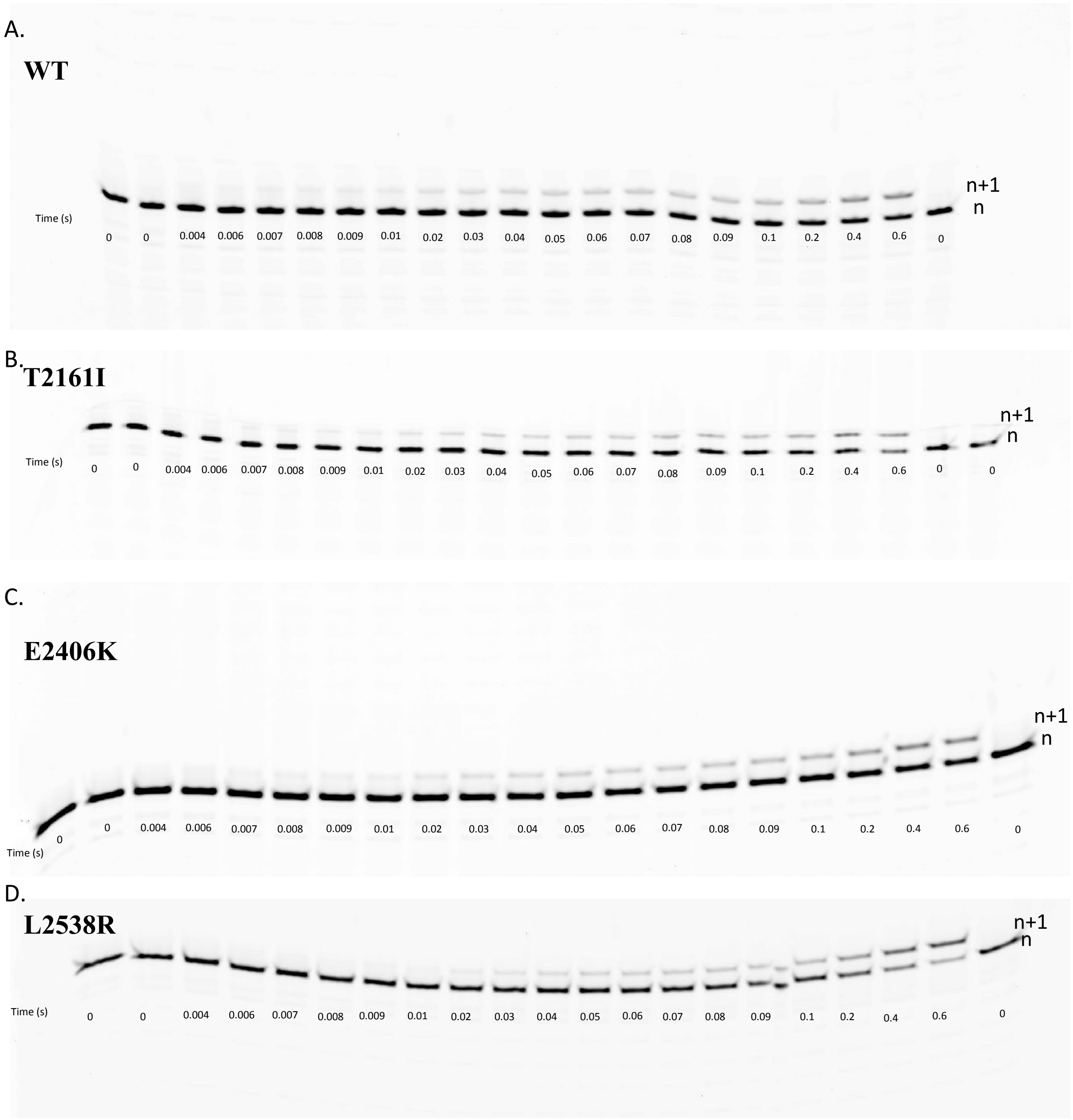

Figure S5. Representative 15% denaturing polyacrylamide gels of pre-steady state burst kinetics for 300 nM WT (A), T2161I (B), E2406K (C), or L2538R (D) and 100 nM duplex DNA. Reactions were incubated with 100 µM dCTP (correct) from 0 to 0.6 s as indicated at 37°C. Samples were loaded with increasing time. A single '0 time point' sample was loaded multiple times on the gel due to inconsistent gel migration. Samples were visualized on an RB Typhoon scanner with FAM filter.

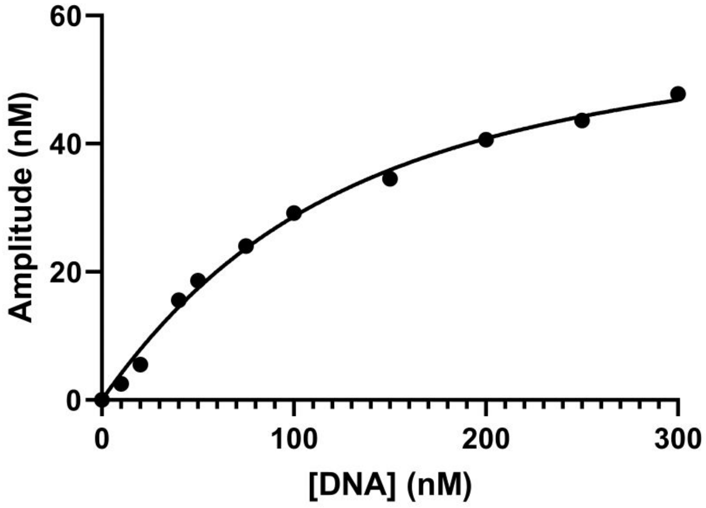

Figure S6. A representative Active site titration demonstrates 62% active protein from one WT protein preparation. Duplex DNA was titrated from 0 to 300 nM against 100 nM Pol θ and 100 μM dCTP 0.003 to 0.6 s. Products were separated and visualized similar to the pre-steady state burst kinetic experiments and an E_{app} was calculated for each DNA concentration. Amplitude versus DNA was graphed to determine K_D(DNA) at 84.1 ± 9.5 nM and Active sites at 62.4 ± 2.7% (± standard error of the fit). Active sites were repeated for each protein

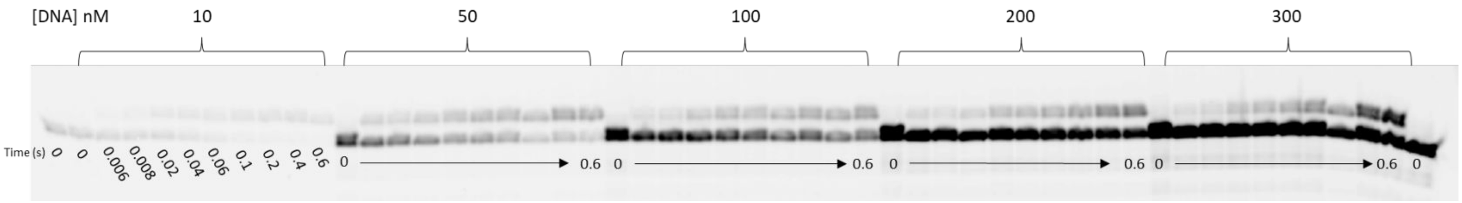


```
Duplex DNA 5'-/FAM/ TTTGCCT TGA CCA TGT AAC AGA GAG
                            CGGA ACT GGT ACA TTG TCT CTC GCA CTC ACT CTC TTC TCT
```

Figure S7. Representative 20% denaturing polyacrylamide gels of WT active site titration. The DNA substrate was titrated from 0 to 300 nM against 100 nM Pol θ and 100 μM dCTP at 37°C from 0-0.6 s. Reactions were stopped by the addition of EDTA and products visualized on an RB Typhoon scanner with FAM filter. A single '0 time point' was loaded multiple times on the gel due to inconsistent gel migration.

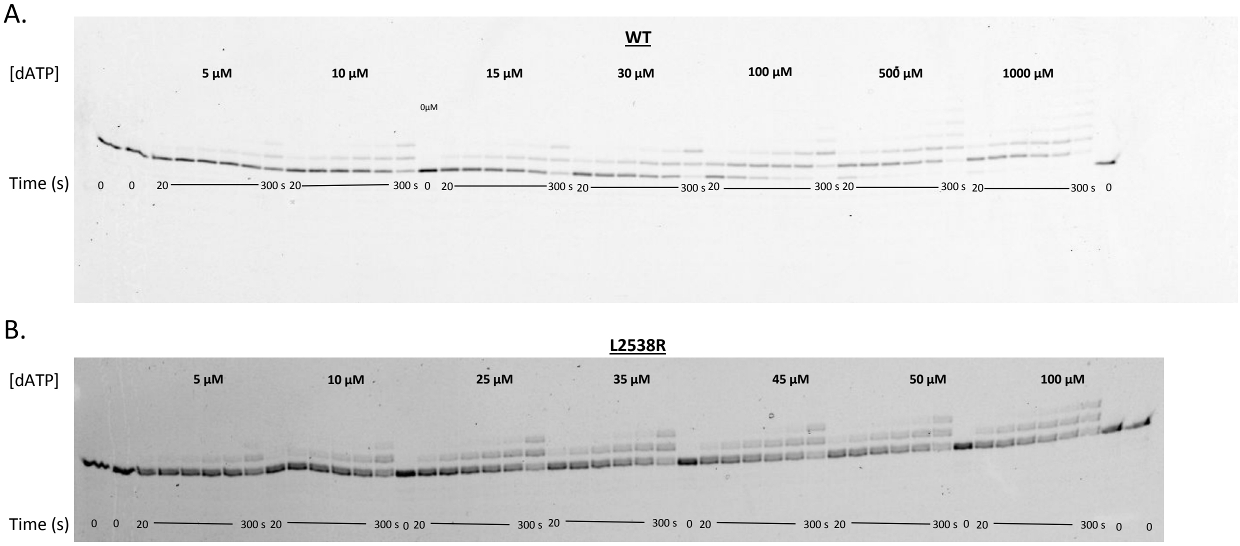


```
Duplex DNA 5'-/FAM/ TTTGCCT TGA CCA TGT AAC AGA GAG
                          CGGA ACT GGT ACA TTG TCT CTC GCA CTC ACT CTC TTC TCT
```

Figure S8. Representative 20% denaturing polyacrylamide gels of incorrect (dATP) single turnover kinetics. Incorrect nucleotide was titrated from 5 µM to 1000 µM against 50 nM duplex DNA and 200 nM WT (A) or L2538R (B) at 37°C from 0-300 s. Reactions were stopped by the addition of EDTA and products visualized on an RB Typhoon scanner with FAM filter. A single '0 time point' was loaded multiple times on the gel due to inconsistent gel migration.

AG DNA 5’-/FAM/ TTTGCCT TGA CCA TGT AAC AGA GAG
CGGA ACT GGT ACA TTG TCT CTC GAA CTC ACT CTC TTC TCT

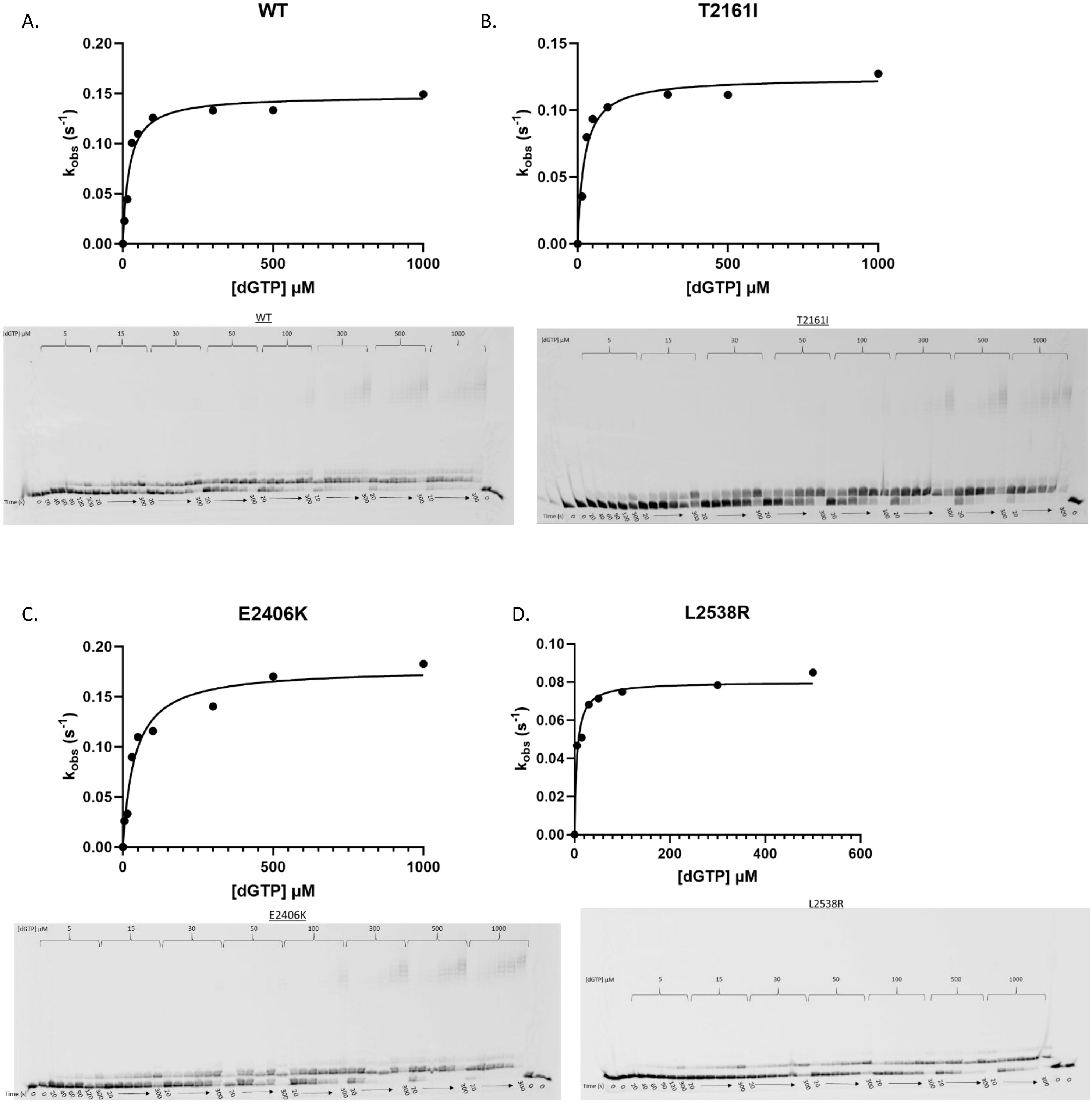

Figure S9. T2161I and L2538R misincorporate dGTP opposite template G with different sequence context. A representative plot and corresponding 15% Native PAGE of single turnover experiments with incorrect dGTP opposite template G with a 5’ template A (highlighted in red) is shown. Increasing concentrations of dGTP (5 µM to 1000 µM) were titrated against 50nM DNA substrate and 200nM WT (A), T2161I (B), E2406K (C) or L2538R (D) for 0-300 s at 37°C. The corresponding representative gel electrophoresis is below each graph. Observed rates versus [dGTP] were plotted and data fit to a hyperbolic equation 5 to determine k_{pol} and $K_{d(dNTP)}$ (± standard error of fit). Results are from three replicates and at least two different protein preparations.

Pol θ	Sequence	k_{pol} (s^{-1})	K_d (μM)	D k_{pol}[a]	ΔD k_{pol}[b]	D K_d[c]	ΔD K_d[d]
WT	G:dC	167	5.20				
T2161I		167	199				
E2406K		89.0	93.1				
L2538R		41.2	15.70				
WT		0.114	1.60	1466		0.31	
T2161I	G:dG	0.223	44.3	746	2	0.22	1
E2406K		0.132	17.4	677	2	0.19	2
L2538R		0.117	3.50	352	4	0.22	1
WT		0.139	9.71	1198		1.87	
T2161I	G:dA	0.133	15.2	1255	1	0.08	23
E2406K		0.160	33.8	557	2	0.36	5
L2538R		0.0920	2.29	449	3	0.15	13
WT		0.150	15.3	1115		2.94	
T2161I	G:dT	0.111	5.50	1507	0.7	0.03	98
E2406K		0.120	10.2	744	1	0.11	27
L2538R		0.125	12.08	329	3	0.77	4

[a]correct/incorrect; [b]WT/variant; [c]incorrect/correct; [d]WT/variant

Table S1. Comparison of discrimination values between WT Pol θ and cancer associated variants.

Pol θ	Sequence	k_{pol} (s^{-1})		$\Delta\, k_{pol}{}^{a}$	K_d (μM)		$\Delta\, K_d{}^{b}$
WT	AG:dG	0.15	± 0.006		21.4	± 4.2	
T2161I		0.12	± 0.006		22.5	± 4.9	
E2406K		0.18	± 0.009		39.9	± 8.5	
L2538R		0.08	± 0.003		5.1	± 1.2	
WT	CG:dG	0.11	± 0.004	1.4	1.60	± 0.4	13
T2161I		0.22	± 0.010	0.6	44.3	± 9.8	0.5
E2406K		0.13	± 0.006	1.4	17.4	± 3.5	2.3
L2538R		0.12	± 0.004	0.7	3.50	± 0.71	1.5

[a]AG:dG/CG:dG; [b]AG:dG/CG:dG

Table S2. Comparison of k_{pol} and $K_{d(dNTP)}$ with an AG template compared to a CG DNA sequence.

REFERENCES

(1) Dalal, S., Chikova, A., Jaeger, J., and Sweasy, J. B. (2008) The Leu22Pro tumor-associated variant of DNA polymerase beta is dRP lyase deficient. *Nucleic Acids Res 36*, 411–422.

(2) Johnson, K. A. (1995) Rapid quench kinetic analysis of polymerases, adenosinetriphosphatases, and enzyme intermediates. *Methods Enzymol 249*, 38–61.
